# Rethinking survey development in health research with AI-driven methodologies

**DOI:** 10.3389/fdgth.2025.1636333

**Published:** 2025-07-28

**Authors:** Hakan Kuru

**Affiliations:** Human-Centered Design, Delft University of Technology, Delft, Netherlands

**Keywords:** artificial intelligence (AI), large language models (LLMs), survey design methodology, ethics, reflexivity

## Abstract

Artificial intelligence (AI), particularly large language models (LLMs), offers new opportunities to address methodological challenges in survey development for health research. Traditional approaches, such as manual item generation, cognitive interviewing, and *post-hoc* psychometric validation, are time- and resource-consuming, and vulnerable to undetected issues that emerge only after large-scale data collection. These limitations, which appear in the early stages, can spread to later phases, leading to costly revisions and weakened construct validity. This paper introduces a conceptual framework for integrating AI-driven techniques throughout the survey development cycles. Drawing on natural language processing, automated text analysis, real-time data monitoring, and predictive modeling, the framework outlines how AI tools can help researchers proactively uncover linguistic nuances, identify hidden patterns, and refine instruments with greater speed and rigor, ultimately enhancing validity, inclusivity, and interpretive richness. Rather than replacing existing practices, these tools are positioned as a complementary support that, when used responsibly and contextually, can enhance methodological rigor, improve efficiency, and reduce respondent burden. The paper also emphasizes ethical considerations, including transparency, interpretability, and mitigation of bias. By combining AI's computational power with human expertise and critical reflexivity, this approach aims to foster more responsive, inclusive, and valid instruments for health-related research and interventions.

## Introduction

Surveys are essential for providing systematic data critical to evidence-based decision-making in health research (1). Traditionally, developing valid and reliable surveys has been a rigorous and multi-step process. The process includes generating candidate items, conducting cognitive interviews to assess comprehensibility, pilot testing the instrument, and applying psychometric methods (e.g., exploratory and confirmatory factor analysis) for the final validation. Beyond the contribution of the quantitative methodology, AI can also enhance qualitative processes, most notably cognitive interviewing and cross-cultural adaptations.

**Figure 1:**
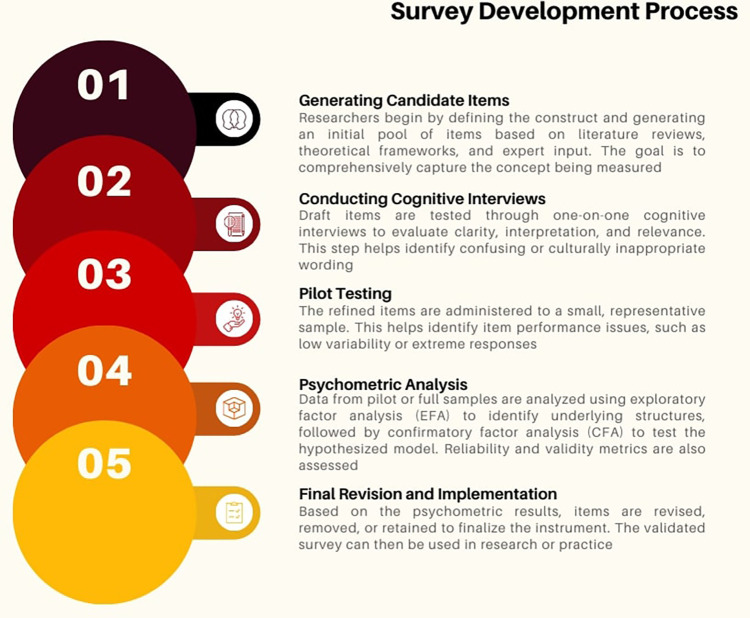
Survey development process.

**Figure 2:**
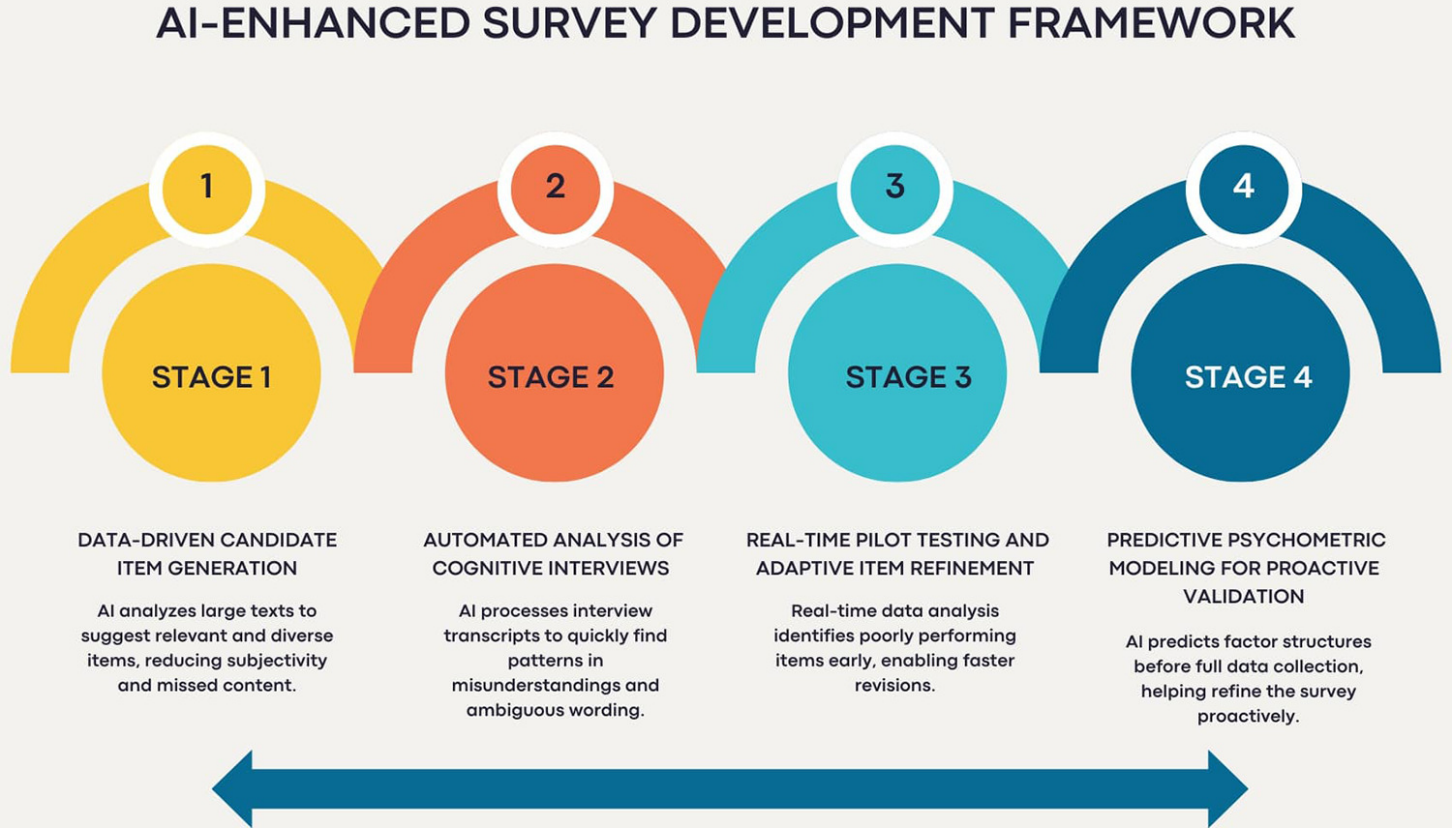
AI-enhanced survey development framework.

**Table 1:** Methodological gaps in survey development.

Survey stage	Traditional approach	Key limitations
Item generation	Literature review and expert judgment	May overlook linguistic nuances or emerging constructs; subject to expert bias
Cognitive interviewing	One-on-one interviews with manual analysis	Time-consuming; small samples; limited capacity to detect widespread interpretation issues
Pilot testing	Small sample testing with *post-hoc* analysis	Issues like floor/ceiling effects only emerge after data collection; difficult to revise efficiently
Psychometric validation	Factor analysis after full data collection	Reveals flaws too late; costly revisions; subject to sample size limitations
Overall development process	Linear and reactive cycle	Problems in early phases often cascade into later stages; inefficient and resource-intensive

**Table 2:** AI enhancement in the survey development process.

Survey stage	AI-based enhancement	Benefits
Item generation	NLP-based thematic analysis of literature and open text sources	Broader item pool; reduced expert bias; detection of latent constructs
Cognitive interviewing	Automated transcription, sentiment analysis, topic modeling	Faster processing; scalable insights into interpretation and wording issues
Pilot testing	Real-time response analytics; anomaly detection	Immediate feedback; adaptive refinement before large-scale testing
Psychometric validation	Predictive modeling to simulate factor structures	Early detection of structural problems; more proactive and efficient validation
Overall development process	Iterative AI-supported feedback loops across all stages	Streamlined survey design cycle; reduced burden; increased validity and inclusivity

The overall methodological limitation of survey development stems from the unpredictability of item clustering before collecting the empirical data. Researchers typically have to wait until a large amount of data has been collected to explore the relationships between items and factors, often resulting in costly and time-consuming iterative revisions (2, 3). LLMs can analyze linguistic and conceptual relationships among survey items even before empirical data collection (4, 5). This enables the early identification of conceptual gaps or clustering patterns, potentially reducing the need for multiple revisions and streamlining the validation process.

The integration of LLMs into survey development has implications. The use of AI tools introduces new ethical considerations, including concerns around bias, transparency, and interpretability (6–8). These aspects must be carefully addressed to ensure responsible and context-sensitive implementation in health research.

This paper explores the potential of AI-assisted pre-factor analysis to enhance the quantitative and qualitative aspects of the survey development process. Accordingly, it reviews the current limitations of traditional methods, presents a conceptual framework for integrating artificial intelligence into survey design, proposes a strategy for pilot implementation, and discusses ethical considerations. In addition, the paper highlights how AI-driven semantic analysis can inform qualitative methods such as cognitive interviewing, ultimately enriching the interpretive rigor and methodological robustness of health research.

## Methodological gaps in survey development

Survey development is a robust and complex process, and even the best practices in each stage present specific challenges that can benefit from more systematic and data-driven methods (see Figure 1).

In the first stage, when generating candidate items for the item pool, the process typically depends on literature reviews, frameworks, and expert judgment (1). Although expert judgment contributes invaluable insight, it may overlook linguistic nuances or emerging themes embedded within a broader context. This can lead to the absence of potentially relevant items, leaving gaps in the representation of the construct within these items (2). More comprehensive analyses can help identify recurring patterns or themes that might be overlooked, ensuring a broader and more accurate representation of the intended constructs.

In the next stage of survey development, qualitative cognitive interviews depend on one-on-one interactions and the manual analysis of participant responses. While these interviews offer deep insights into how respondents understand and respond to each survey item, they are often time-consuming and involve a limited number of participants (9, 10). Since this process relies on manual analysis, it could be challenging to detect misunderstandings across large groups. In many cases, nuanced differences in interpretation may be overlooked because the qualitative interview process does not effectively explore these aspects. Employing more systematic and quantitative methods for analyzing interview transcripts could uncover problematic issues in wording or comprehension more effectively.

During the pilot testing phase, surveys are administered to a relatively small sample to evaluate the preliminary performance of items. Problems such as unexpected response patterns, floor or ceiling effects, or inconsistent item performance are often not recognized until after data collection, necessitating additional revisions (1, 3). This reactive approach slows down the development cycle and consumes additional resources. Techniques that analyze response data more quickly could provide real-time insights into item performance, allowing adjustments before large-scale implementation.

Finally, in the psychometric validation phase, standard methods such as exploratory and confirmatory factor analysis are applied only after a complete dataset is available. While these techniques are essential for verifying construct validity (11, 12), they also highlight structural problems late in the process, often requiring extensive revisions. These challenges are further complicated by practical constraints, such as limited sample sizes resulting from logistical or ethical factors (9). Poorly performing items can undermine the instrument's effectiveness. And necessitate removal or further data collection, increasing costs and extending timelines. Proactive and predictive approaches could anticipate such issues earlier, leading to more efficient validation (see Table 1).

Importantly, problems that originate in early stages, such as vague item wording or undetected ambiguities during cognitive interviews, often cascade into later phases of development. For example, suppose a construct is poorly represented in the item pool or misinterpreted during pretesting. In that case, this flaw might remain hidden until psychometric validation, at which point fixing it would require a major redesign and re-administration. These downstream effects increase time, cost, and participant burden. A more anticipatory, AI-supported approach could help surface such issues earlier and prevent costly revision cycles.

Addressing these methodological gaps requires a proactive approach to identifying and resolving potential survey design issues early, resulting in a more streamlined validation process and a more reliable final instrument.

## Advancing survey development through AI

Recent advances in Large Language Models (LLMs) offer an innovative opportunity to improve the survey development process (4). LLMs provide targeted solutions to methodological gaps and enhance each stage of the process. During candidate item generation, traditional methods rely on manual literature reviews and expert judgment, which may miss linguistic nuances or emerging themes. In contrast, leveraging techniques from natural language processing (NLP) enables the systematic scanning of large bodies of text, such as academic journals, reports, and historical surveys, to detect recurring keywords, conceptual overlaps, and nuanced language patterns. These methods can help generate a more comprehensive and representative item pool, reducing subjectivity and ensuring that critical aspects of the constructs are fully captured (1, 13, 14).

In the stage of qualitative cognitive interviews, the traditional reliance on one-on-one interactions and manual coding of interview transcripts often results in a time-consuming process with a limited scope. Computational text analysis can transform this stage by automatically transcribing and coding large volumes of interview data, revealing patterns in respondent interpretations. For example, automated sentiment analysis and topic modeling methods can identify recurring misunderstandings or ambiguities in item wording across numerous interviews, capturing nuances often overlooked in manual analysis. This systematic approach accelerates data processing and enhances the consistency and depth of the qualitative evaluation (9, 10, 15).

During pilot testing, surveys are traditionally administered to a small sample, with issues such as unexpected response patterns or extreme scoring effects identified only after data collection. Advanced analytics techniques can monitor responses in real-time, flagging problematic items such as those with extreme responses or inconsistent patterns at an early stage. This immediate feedback enables quick adjustments to the survey design before full-scale deployment, thereby reducing the need for multiple testing cycles (1, 3). Additionally, modern predictive modeling techniques can dynamically assess item performance, leading to faster, evidence-based refinements.

Finally, in the psychometric validation phase, exploratory and confirmatory factor analyses often reveal problems after extensive data collection. By applying computational modeling techniques earlier, researchers can simulate and predict potential factor structures in advance. These predictive analyses can highlight problematic items and suggest structural adjustments in advance, reducing the risk of costly revisions. This approach supports a more efficient and robust validation process (11, 12, 16).

## AI-enhanced survey development: a conceptual framework

In modernizing the survey development process, a conceptual framework that integrates advanced computational techniques offers a clear and structured approach to addressing existing methodological gaps. The framework outlines how AI tools can work in conjunction with traditional methods at every stage of survey development, ensuring that each step benefits from objective, scalable, and timely support.

### Stage 1: data-driven candidate item generation

Survey development typically begins with generating a pool of items based on expert opinion and literature (1). However, by using AI techniques, researchers can scan large amounts of textual data from academic papers or existing surveys to detect commonly used terms, patterns, and themes. This helps generate a more complete and accurate list of items, reducing bias and increasing the likelihood that all crucial aspects of a topic are included (13, 14).

### Stage 2: automated analysis of cognitive interviews

After creating the initial item pool, researchers often conduct cognitive interviews to verify that people understand the questions as intended. Traditionally, these interviews involve labor-intensive interactions and manual coding (9, 10). With automated transcription and text analysis, large volumes of interview data can be processed rapidly and consistently. This enables researchers to quickly identify unclear wording, misunderstandings, or cultural issues that may influence how respondents interpret survey items (15).

### Stage 3: real-time pilot testing and adaptive item refinement

Pilot testing is used to evaluate the performance of an item in a small group before its full-scale use. Traditional methods often require waiting until data collection concludes to identify issues such as floor or ceiling effects (1, 3). The framework, however, can monitor responses as they are collected, identifying problems such as confusing items or strange response patterns. This means issues can be fixed earlier, speeding up the process and improving data quality.

### Stage 4: predictive psychometric modeling for proactive validation

Survey validation typically relies on exploratory and confirmatory factor analysis after the collection of complete data (11, 12). The framework introduces predictive modeling to simulate factor structures in advance. These models forecast item performance, anticipate issues like weak factor loadings or misalignments, and recommend structural refinements. This proactive analysis serves as an early warning system, enabling researchers to avoid surprises and make necessary adjustments earlier in the process (16).

At the heart of this framework is the concept of continuous feedback loops. Insights from candidate item generation, automated cognitive interview analysis, real-time testing, and predictive modeling feed back into the development cycle. This iterative refinement ensures the survey evolves to be both valid and respondent-centered (see Figure 2).

## Discussion

This manuscript presents a pioneering exploration of integrating artificial intelligence (AI), particularly large language models (LLMs), as transformative tools in health research, with a specific focus on survey development processes. The integration of AI addresses key methodological challenges, such as inherent subjectivity in item generation, scalability issues in cognitive interviewing, delayed identification of design problems during pilot testing, reactive psychometric validation practices, and constraints imposed by small sample sizes. When carefully applied, AI techniques may offer valuable support in improving the rigor and efficiency of survey development research (1–3) (see Table 2).

AI-driven techniques can help reduce subjective bias during item generation by identifying linguistic and thematic patterns through natural language processing (NLP). Combined with traditional expert input, this approach may enhance construct representation by incorporating broader semantic dimensions (6, 13, 14). However, the quality and relevance of AI outputs depend on the quality and relevance of the input data, as well as the contextual fit with the research domain.

The manuscript introduces the use of automated text analytics in cognitive interviews. Sentiment analysis and topic modeling can support faster data processing and may reveal interpretative patterns that manual analysis might overlook. Yet, these methods should be interpreted carefully and triangulated with human judgement to ensure cultural sensitivity and analytical validity (10, 15).

Incorporating predictive analytics into pilot testing represents a significant methodological opportunity. AI tools can help detect problematic items earlier, potentially reducing iterative revision cycles (1, 3). Nonetheless, the accuracy of such feedback is closely tied to the representativeness of pilot data and the reliability of the underlying models.

Predictive psychometric modeling enables anticipatory analysis of factor structures, which may assist in refining item pools before large-scale data collection. These tools offer a way to anticipate weak factor loadings or conceptual drift. However, their utility must be evaluated in relation to theoretical frameworks, sample diversity, and model transparency (11, 12, 16).

Despite these advances, this manuscript also addresses essential ethical and methodological considerations in the integration of AI. Biases in training data can compromise fairness and representativeness. Thus, the need for strong ethical frameworks and interdisciplinary collaborations is emphasized (17–19).

### Reflexivity and ethical considerations

Reflexivity, a cornerstone of health research, entails critically examining the researcher's role, assumptions, and influence on the research process (20). Reflexivity becomes even more vital when integrating artificial intelligence (AI) methods into survey development. Researchers must actively interrogate how their views on technology, efficiency, and innovation shape the design, analysis, and interpretation processes. While this manuscript advocates for the strategic use of Large Language Models (LLMs) to enhance qualitative rigor and scalability, it is essential to acknowledge the underlying technology that may influence these choices.

Moreover, the reliance on AI-driven methods introduces complex layers of bias and limitation. LLMs are trained on extensive human-generated corpora that inevitably reflect dominant sociocultural narratives and systemic biases (6). Consequently, automated analyses may unintentionally marginalize less dominant or culturally specific health experiences, reinforcing inequities in research. Researchers must remain aware that algorithmic outputs are not neutral but are products of historical and cultural data imprints, necessitating careful validation within diverse health contexts.

Transparency and ethical responsibility are paramount when utilizing AI in research. Researchers should document the selection of AI tools, the data processing steps, and how the outputs were incorporated into analytic processes (7). Additionally, data privacy, participant consent, and reidentification risks associated with automated text analysis necessitate explicit ethical safeguards (18). As many AI ethics frameworks are still evolving, researchers should proactively establish and uphold ethical standards.

To address these challenges, several reflexive strategies are recommended. First, AI-generated findings should be triangulated with human-led thematic analyses to ensure cultural sensitivity and interpretive depth. Second, involving diverse stakeholders in interpreting AI-identified patterns can mitigate bias and strengthen validity. Third, ongoing human reviews of AI outputs should be embedded throughout the research process to maintain responsiveness to meaning and context. Researchers must continuously ask what AI reveals and what it might obscure or misrepresent.

Ultimately, AI should be positioned as a tool that supports rather than replaces human interpretive judgment in research. By foregrounding reflexivity and ethical scrutiny, researchers can harness the innovative potential of AI while preserving the core principles of empathy, contextual awareness, and critical inquiry. This commitment is essential for the responsible advancement of research.

## Call to action and future directions

Given these insights, the manuscript strongly advocates for researchers to proactively embrace and explore AI methodologies in their research. To move from concept to application, future research must establish rigorous methodological protocols and best-practice standards for AI integration within research, emphasizing transparency and reproducibility. This includes developing methodological protocols that document how AI tools are selected, how data are processed, and how AI outputs are interpreted in the context of theory. Without such protocols, reproducibility and scientific accountability may be compromised, particularly in high-stakes health contexts.

Additionally, research should focus on developing adaptive and context-sensitive AI tools that enhance data collection and participant engagement. Instead of generic algorithms, AI systems used in survey development should account for linguistic variation, cultural context, and domain-specific knowledge. Doing so would enhance the inclusiveness and relevance of survey instruments, especially in global or multilingual research settings.

Ethical frameworks must also evolve in parallel with technical innovation. AI systems used in research carry inherent risks of reinforcing bias, amplifying inequity, or compromising privacy. Future work should develop mechanisms to identify and monitor these risks across the survey development lifecycle. This includes participatory design, diverse stakeholder involvement, and embedding human review loops throughout the AI pipeline.

Ultimately, future studies should evaluate the impact of AI-driven methods on long-term research outcomes, encompassing not only survey quality but also knowledge production, equity, and decision-making. Longitudinal evaluations will be essential to understand how these tools influence the validity and usefulness of the data they help produce. In turn, this evidence can inform best practices and policy recommendations for the responsible use of AI in the behavioral and health sciences.

## Conclusion

This manuscript contributes a novel and scientifically grounded approach for integrating AI into research, particularly in survey development. Rather than replacing existing methods, AI tools should be viewed as complementary resources that, when properly implemented, can help address specific methodological challenges. However, these benefits are not automatic and require careful design, interpretation, and oversight. The proposed framework demonstrates how AI tools can be strategically applied across the survey lifecycle to reduce resource burden, accelerate development, and enhance construct validity.

To realize this potential, interdisciplinary collaboration and critical reflexivity are essential. Researchers, data scientists, and ethicists must collaborate to ensure that AI applications are both effective and socially responsible. With careful attention to design and implementation, AI can help move research toward a more responsive, inclusive, and innovative future.

## Data Availability

The original contributions presented in the study are included in the article/Supplementary Material, further inquiries can be directed to the corresponding author.
